# Assessing the Feasibility of Controlling *Aedes aegypti* with Transgenic Methods: A Model-Based Evaluation

**DOI:** 10.1371/journal.pone.0052235

**Published:** 2012-12-21

**Authors:** Mathieu Legros, Chonggang Xu, Kenichi Okamoto, Thomas W. Scott, Amy C. Morrison, Alun L. Lloyd, Fred Gould

**Affiliations:** 1 Department of Entomology, North Carolina State University, Raleigh, North Carolina, United States of America; 2 Department of Entomology, University of California Davis, Davis, California, United States of America; 3 Division of Earth and Environmental Sciences, Los Alamos National Laboratory, Los Alamos, New Mexico, United States of America; 4 Fogarty International Center, National Institutes of Health, Bethesda, Maryland, United States of America; 5 Department of Mathematics and Biomathematics Graduate Program, North Carolina State University, Raleigh, North Carolina, United States of America; University of Texas Medical Branch, United States of America

## Abstract

Suppression of dengue and malaria through releases of genetically engineered mosquitoes might soon become feasible. *Aedes aegypti* mosquitoes carrying a conditionally lethal transgene have recently been used to suppress local vector populations in small-scale field releases. Prior to releases of transgenic insects on a wider scale, however, most regulatory authorities will require additional evidence that suppression will be effective in natural heterogeneous habitats. We use a spatially explicit stochastic model of an *Ae. aegypti* population in Iquitos, Peru, along with an uncertainty analysis of its predictions, to quantitatively assess the outcome of varied operational approaches for releases of transgenic strains with conditional death of females. We show that population elimination might be an unrealistic objective in heterogeneous populations. We demonstrate that substantial suppression can nonetheless be achieved if releases are deployed in a uniform spatial pattern using strains combining multiple lethal elements, illustrating the importance of detailed spatial models for guiding genetic mosquito control strategies.

## Introduction

Application of transgenic strategies to manipulate mosquito species that transmit malaria and dengue has attracted considerable scientific and media attention [1]–[3]. Genetically-modified mosquitoes could be used for two main strategic purposes: population suppression (lowering densities, and ideally eliminating, local vector populations) or population replacement (replacing resident competent vectors with transgenic strains that do not contribute to pathogen transmission).

Before genetically-modified mosquitoes can be released in the environment, regulatory authorities and funding agencies require risk analyses. Approaches aimed strictly at population suppression offer a favorable profile with regard to some environmental risks because the transgenes are predicted to be lost from populations after releases are ended [4], whereas population replacement strains that include self-propagating genetic elements face concerns associated with design features enabling them to persist in the environment [5], [6].

Dengue causes more human morbidity and mortality than any other mosquito-borne virus with an estimated 50 million humans infected and 500,000 cases of life-threatening dengue hemorrhagic fever (DHF) and dengue shock syndrome (DSS) each year [7], [8]. Unlike malaria and other mosquito-borne diseases, dengue has only one major vector species, *Aedes aegypti*. Although other *Aedes* species can be involved, their role in disease transmission is minor [9]. *Ae. aegypti*, in addition, is easier to rear and genetically engineer than malaria vectors. This species has therefore become an important target for new genetic approaches for vector control.

An *Ae. aegypti* strain designed for population suppression based on the use of a dominant genetic element that kills male and female offspring was the first genetically modified mosquito to be released into the environment [2], [10]. The report of the results of this trial generated lively debate in scientific and general media [2], [3], demonstrating that the release of genetically-modified mosquitoes in natural environments remains a sensitive and controversial issue, and requires a cautious series of steps in transgenic strain testing. For strains using a dominant lethal genetic element, and strains using a similar genetic element that kills only female offspring, potential efficiency has been examined using general mathematical models [11]–[13] which all conclude that release of such strains will cause elimination of local mosquito populations.

Moving from these modeling results and laboratory experiments [14] to large scale field releases in cities remains a far from trivial endeavor. Experience of early workers who tried to use genetic techniques for mosquito control demonstrated that even results from outdoor field cages cannot accurately predict the success of a genetic control strain in the pest’s heterogeneous natural habitat [15]. General mathematical models, field cage trials, and even small scale open releases fail to account for the effects of a number of environmental factors on mosquito dynamics, including spatial heterogeneity in mosquito density, adult dispersal, and interactions with human populations.

Because strategies for releasing transgenic mosquitoes into large, heterogeneous populations cannot be investigated with general models that make simplifying assumptions regarding the structure and dynamics of the target population, we developed the Skeeter Buster model [16], a stochastic, spatially explicit simulation model of *Ae. aegypti* populations, with the specific objective of examining the merits of different approaches for dengue prevention, particularly in heterogeneous environments. Herein, we use Skeeter Buster to compare distinct intervention strategies based on a recently engineered genetically modified mosquito strain [17]. In this strain, a transgene for a toxic protein is controlled by a promoter that only turns on expression in female flight muscles. Females with dead muscle cells cannot fly, and consequently cannot feed or mate, and die soon after emergence.

This sex-specific lethality strategy, hereafter referred to as female killing (FK), is particularly attractive in part because it allows population suppression effects to be carried through multiple generations by viable male offspring, particularly if several independent lethal elements are introduced [18]. From a more practical standpoint, female-specific adult killing is attractive because (i) it provides an easy means of releasing only male mosquitoes into the environment–desirable because only adult females take blood meals and transmit virus–and (ii) targeting adults allows transgene bearing individuals to remain viable throughout immature stages, therefore acting as competitors against wild-type larvae, and maximizing the reduction in the number of wild-type adults emerging from breeding sites (whereas early removal of transgenic offspring could lead to improved breeding site conditions for the remaining wild-type larvae, potentially resulting in little or no reduction in the number of adults) [12], [19], [20].

The authors reporting on construction of this FK strain propose a deployment method where eggs would be shipped throughout a city. Local, community stakeholders would then be responsible for hatching eggs in suitable habitats so that FK adults would emerge but only males would be viable. These males would then mate with wild-type females, whose female progeny would not develop into viable, virus-transmitting adult females [17]. *Ae. aegypti* is particularly amenable to this approach because its eggs are desiccation resistant and can remain viable for several months, making them easy to store, ship, distribute, and hatch simply by immersing in water. This would require a high level of community involvement, which has long been recognized as desirable for vector control programs [21]–[23] and is considered an important component of global dengue control efforts [24]–[27]. In our study, we use the Skeeter Buster modeling tool to compare the use of egg releases to the release of adult mosquitoes.

Skeeter Buster is a complex, biologically-detailed model that tracks the development of cohorts of immature mosquitoes in individual breeding sites, as well as individual adults in houses. Specific aspects of life history are simulated at each life stage (see Methods and reference [16] for further details), including notably temperature-dependent survival rates, water level fluctuations influencing egg hatching, and nutritional contents of individual breeding sites influencing (in a density-dependent fashion) larval development time and survival.

Because of this level of detail, the interpretation of simulation results is contingent on the underlying biological assumptions in the model, as well as model parameterization. The latter involves several calibration steps to perform simulations in a location specific fashion, based on data regarding local weather as well as distribution and characteristics of breeding sites in the location of interest. In this study we simulate releases in the Amazonian city of Iquitos, Peru, building on prior success of simulating the equilibrium population dynamics of *Ae. aegypti* at this location [28].

In the main text of this paper, we present results for the case where transgenic mosquitoes have no fitness cost and have a mating competitiveness equal to that of wild-type individuals. If fitness and mating costs exist, the main effect would be that more mosquitoes would need to be released to achieve a similar level of population reduction (see selected scenarios in Fig. S1).

Because the model is stochastic, we simulate each scenario 30 times. As a benchmark for success of a particular approach, we examine the status of the population, reporting whether local elimination of the resident population has been achieved or not, after 3 years of control. Although a longer time frame might enhance the probability of success, community involvement and resource allocation are likely to wane over a 3-year time period. As described in our methods, we simulate combinations of three different spatial release patterns (release into 100% of premises, or 10% randomly or uniformly distributed premises), two different life stages that could be released (eggs or adults), and two types of FK strains (one or three independently assorting FK construct insertions). We test the hypothesis that spatial heterogeneity in the vector population will be an important challenge for release strategies to address and illustrate risks associated with decreasing community participation as the release program advances through time. Results from an uncertainty analysis identify traits of *Ae. aegypti* life history and ecology that have the largest impact on model predictions, indicating which refined empirical estimates would be most beneficial to improve model guidance of large-scale FK-based control programs.

## Results

### Homogeneous vs. Point Source Release of FK Male Adults

We first simulate the release of FK male adults in our model area. Skeeter Buster models individual premises (in this context, a premise represents a household in the city, including indoor and outdoor parts of the property) laid out on a rectangular grid that generally resembles the neighborhoods of Iquitos. Adult mosquitoes can disperse within the grid from one premise to one of its neighbors (see Methods) but immigration from adjacent populations into our simulated population is ignored. We consider weekly releases, with two main types of spatial distribution: (i) homogeneous release, in which FK males are released uniformly in or next to every premise in the city and (ii) point source release, in which FK males are released at a limited number (242, ie. approximately 10%) of uniformly or randomly selected premises. When a household is randomly selected as a release site, it is used throughout the simulation. This reflects the real-world situation in which about 10% of households agree to become cooperators. Releases could be accomplished by air, or with land vehicles driving along streets. When a limited number of release sites must be chosen, decisions regarding their spatial distribution will likely be affected by a number of logistical constraints. In this study we examine the impact of this distribution by modeling two simple cases: uniform and random distribution of release sites. Although actual releases are unlikely to exactly mimic the insect distributions in our simulations, the range of scenarios examined with the model provides insights about the impacts of diverse field release approaches.

**Figure 1:**
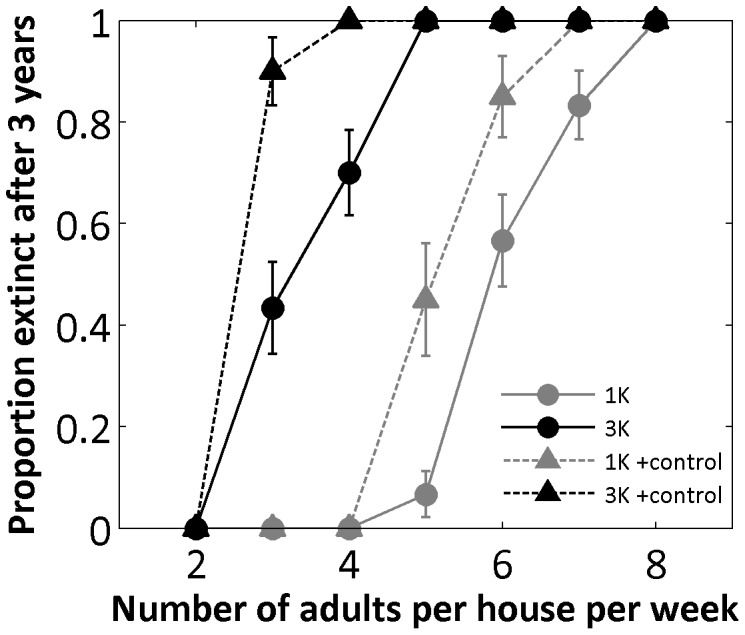
Figure 1. Adult male releases in each house (homogeneous strategy). Proportion of simulations that reach extinction after 3 simulated years when males are released weekly. For each scenario 30 replicated simulations were run. Solid lines and circles: simulations without pre-release control; dashed lines and triangles: simulations with 2-week pre-release insecticidal control. Gray lines: 1 lethal element (1 K); black lines: 3 lethal elements (3 K). Error bars show estimated proportion (*p*) +/− standard error (calculated using 
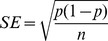
, with *n* = 30 replicates).

**Figure 2:**
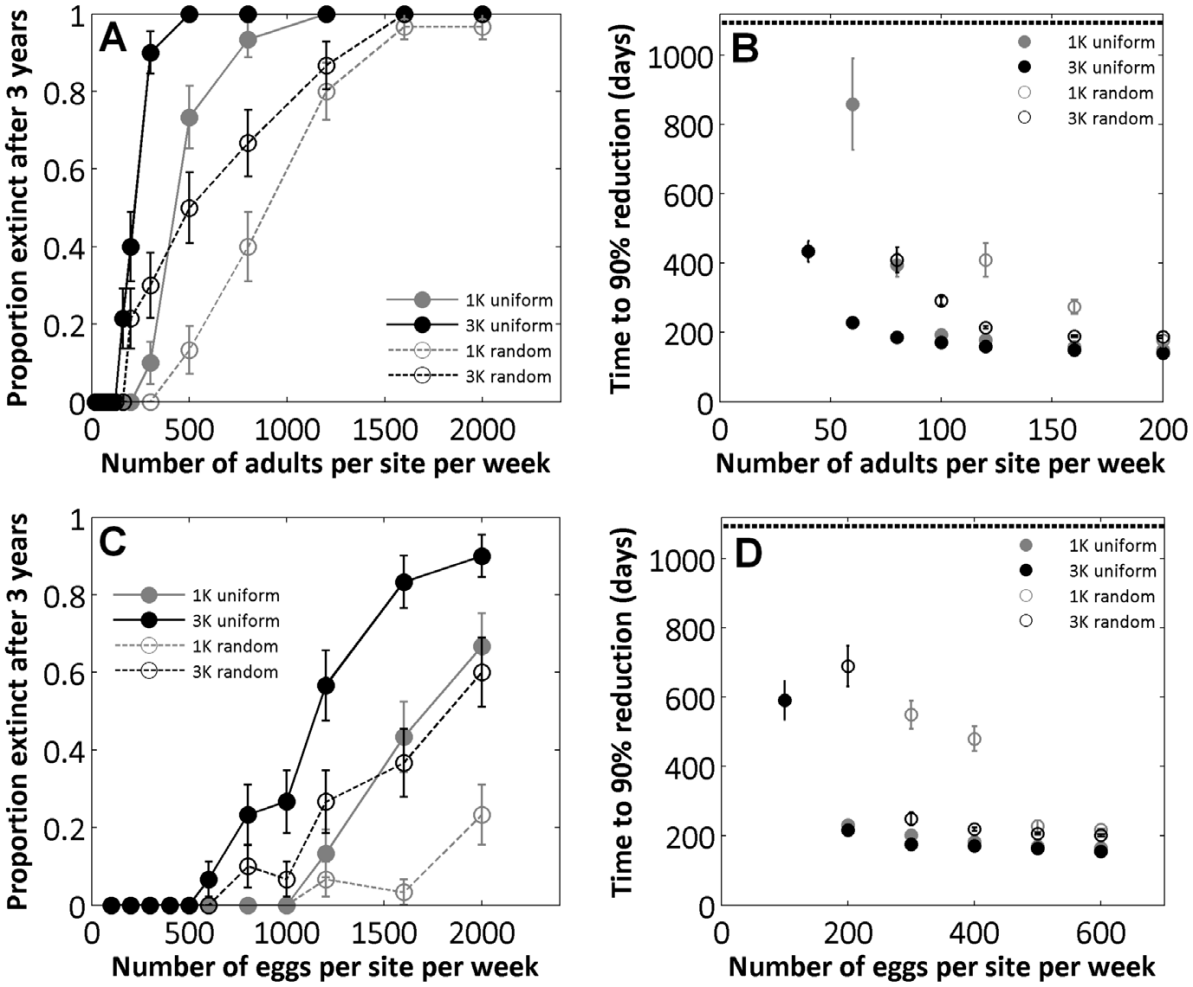
Figure 2. Releases in 10% of houses. Proportion of simulations that reached extinction after 3 simulated years (left panels A and C) and time to reach 90% population reduction (right panels B and D) for point source release strategies, when male adults (upper panels A–B) or eggs (bottom panels C–D) are released weekly. For each scenario 30 replicated simulations were run. Solid lines and filled markers: uniform distribution of release sites; dashed lines and open markers: random distribution of release sites. Gray lines: 1 lethal element; black lines: 3 lethal elements. A–C: Error bars give the estimated standard error as in Fig. 1. B–D: error bars give standard deviation. Note that in panels B and D, individual simulations that do not reach at least a 90% reduction in 3 years (horizontal line) were excluded from calculations of the average and SD.

Figure 1 illustrates the relationship between the number of homogeneously released males and the proportion of simulations in which elimination was achieved within 3 years in the modeled 2448 house area. If at least 8 males of a strain with one FK construct are released per house weekly, elimination occurs in all simulations. For a strain with three independently segregating FK constructs (noted 3 K) only 5 males per house are required. A typical house in the Iquitos simulations (reflecting densities adjusted to field surveys from 1999–2003 [28]) had approximately 2 naturally occurring adult males per house. This release level therefore constitutes an approximate initial release ratio of 2.5∶1. In contrast, point source releases to 10% of houses require release of at least 400 adult FK males per week per site to achieve elimination in all 30 simulation runs when the release locations are uniformly spaced from each other. At least 1200 FK males are required per week if sites are randomly located (Fig. 2A). These numbers per release site per week, respectively correspond to initial release ratios of 20∶1 and 60∶1 for each of the 2448 houses.

For point source releases, we also measured the time required for the population to be reduced by 90% (Fig. 2B). When the release sites are spaced at a uniform distance from each other, the densities decline to a 90% reduction more quickly than when sites are randomly distributed. In cases with randomly located sites, at least 60 FK males with 3 lethal elements or 120 FK males with 1 lethal element are needed per site and per week to achieve a 90% reduction within 3 years. In all simulation comparisons, the strain with 3 constructs is substantially more efficient than the strain with 1 construct (Figure 2A and 2B).

### Release of FK Eggs

Next, we simulated the release of FK eggs in the modeled area. Under this scenario, point source release is the only feasible option as described by Fu et al. [17]. As in the case of point source release of adults at 10% of sites, elimination was more likely when release sites are uniformly distributed. Under the uniform spatial distribution, at least 1600 eggs of the strain with three elements must be released per site per week to obtain elimination in more than 80% of simulations (Fig. 2C). Under the optimal nutrient conditions expected in the simulated release containers [17], [29], 1600 eggs result in approx. 600 adult males (and no viable females) emerging over a period of 2 to 3 days. Reaching 90% population reduction can be achieved with release numbers as low as 200 eggs for the strain with three lethal elements (Fig. 2D). Here again, the uniform distribution of release sites facilitates reaching the goal of 90% reduction, and the strain with three lethal elements is more effective across all scenarios.

### Importance of Pre-release Traditional Control

Because the efficacy of all SIT (Sterile Insect Technique) approaches, including FK strategies, depends on the ratio of released to resident males, SIT programs are typically initiated when the resident population density is lowered by a traditional, density-independent control method or during seasonally related low density. Because the population dynamics of *Ae. aegypti* in Iquitos do not exhibit marked seasonality, FK releases would most likely follow a period of mosquito population reduction efforts by traditional methods such as insecticide spraying. We simulate a highly effective control program with a low residual insecticide during a 2-week period by adding an additional 90% daily mortality probability to every adult in the targeted area during that period. The result of this pre-release reduction is that about 15–25% fewer FK males are needed per week to achieve extinction in 100% of the simulations (Fig. 1).

### Effects of Spatial Heterogeneity in the Vector Population

**Figure 3:**
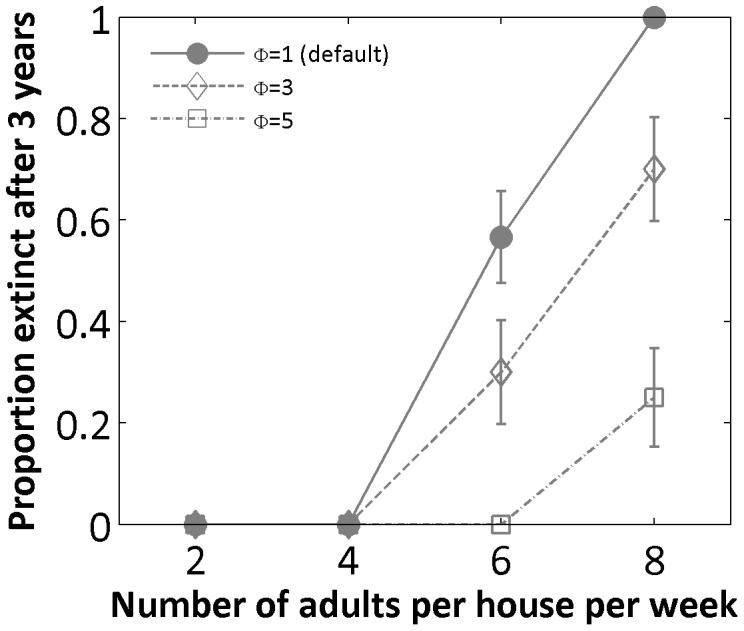
Figure 3. Increased resident heterogeneity. Proportion of simulations that reach extinction after 3 simulated years when male adults carrying 1 lethal element were released in every house (homogeneous strategy) into a population with increased heterogeneity in container distribution. For each scenario 30 replicated simulations were run. Circles: unchanged distribution *Φ* = 2; diamonds: increased heterogeneity *Φ* = 3; squares: increased heterogeneity *Φ* = 5. Error bars give the estimated standard error as in Fig. 1.

FK releases that distribute FK males homogeneously across all houses or release them uniformly in point sources are always predicted to be more efficient than releases in randomly selected point sources, but fail nonetheless to achieve extinction in a wide range of scenarios. This failure occurs because, in local clusters of highly productive houses, the ratio of FK adults to resident wild-types is locally lower than average, reducing the efficacy of population suppression in these specific clusters. Considering that many dengue-endemic cities can exhibit higher heterogeneity than Iquitos, which shows moderate levels of heterogeneity among houses [30], [31], it is important to characterize how FK strategies would fare in the face of increased levels of heterogeneity. We show that in a scenario with release of FK-bearing adult males in every house throughout our modeled area, the probability of achieving elimination is significantly reduced if the variability among houses is increased (Fig. 3). The time required to reach a 90% population reduction is also increased when spatial heterogeneity is higher, but the impact is less compared to the probability of elimination (Fig. S2).

### Impact of Early Male Dispersal

**Figure 4:**
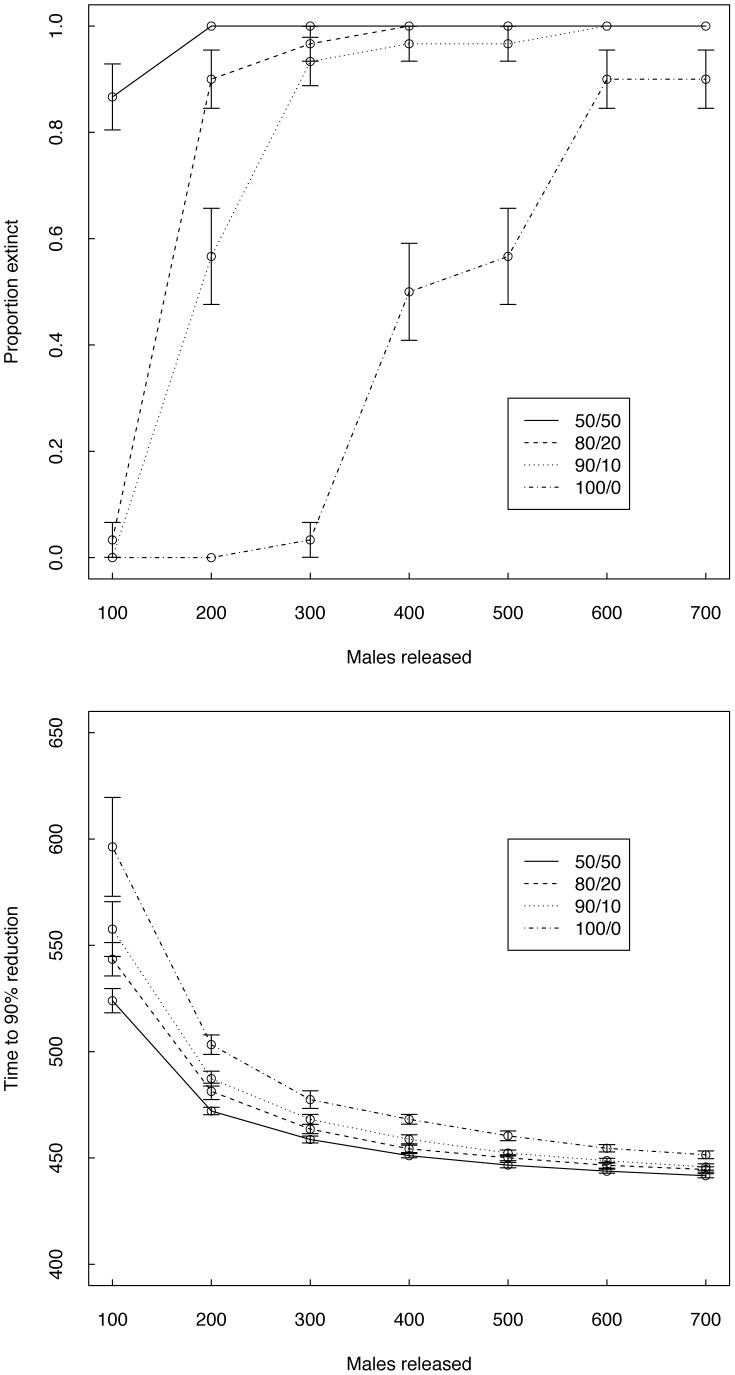
Figure 4. Impact of early dispersal of adult males. Male adults (carrying 1 FK construct) are released from 10% uniformly distributed release sites into a resident population with natural heterogeneity. The first number in the legend indicates the percentage of the released cohort that is assigned to the specific release site. The second number indicates the remaining percentage of released adults assigned on release to a random site in the grid. Top panel: elimination probability; bottom panel: time to 90% reduction.

When large numbers of adult mosquitoes are released in a point source, a fraction is expected to disperse immediately upon release [32], beyond the typical daily dispersal incorporated into the model. We investigated the impact of such early dispersal on strategies involving the release of adult males in 10% of (uniformly distributed) release sites. In these simulations, only a fraction of released males stayed in the designated release site on the day of release, whereas each remaining adult was assigned to a different house chosen at random within the entire simulated area (Valerio et al. [32] found that the median distance moved within 2-days was only between 10 and 50 meters but a small fraction moved more than 100 meters). We show that this early dispersal facilitates population elimination (Fig. 4). With as low as 20% early dispersers in a release cohort, the number of males to release to achieve elimination in 90% of the simulations is lowered from 600 to 200. Similarly, the time required to achieve a 90% reduction in population density is lowered when the fraction of early dispersers increases (Fig. 4).

### Effects of Decreasing Participation Over Time

Point source releases from selected premises by community members offer several practical advantages that make them attractive approaches from an operational standpoint [17]. A potential problem with community involvement in dengue suppression programs, however, is declining community participation as a control program goes on [33]. This is a particularly challenging threat to long-term control programs like SIT- or FK-based approaches. We show that a decrease in participation can have important effects on the level of population reduction in the city, in some cases bringing the population levels back up to 65% of the pre-intervention levels (Fig. S3). The effect of a similar decrease in participating release sites is more dramatic when release sites are initially randomly versus uniformly distributed (Fig. S3), further arguing for careful selection of release sites.

### Uncertainty Analysis

**Figure 5:**
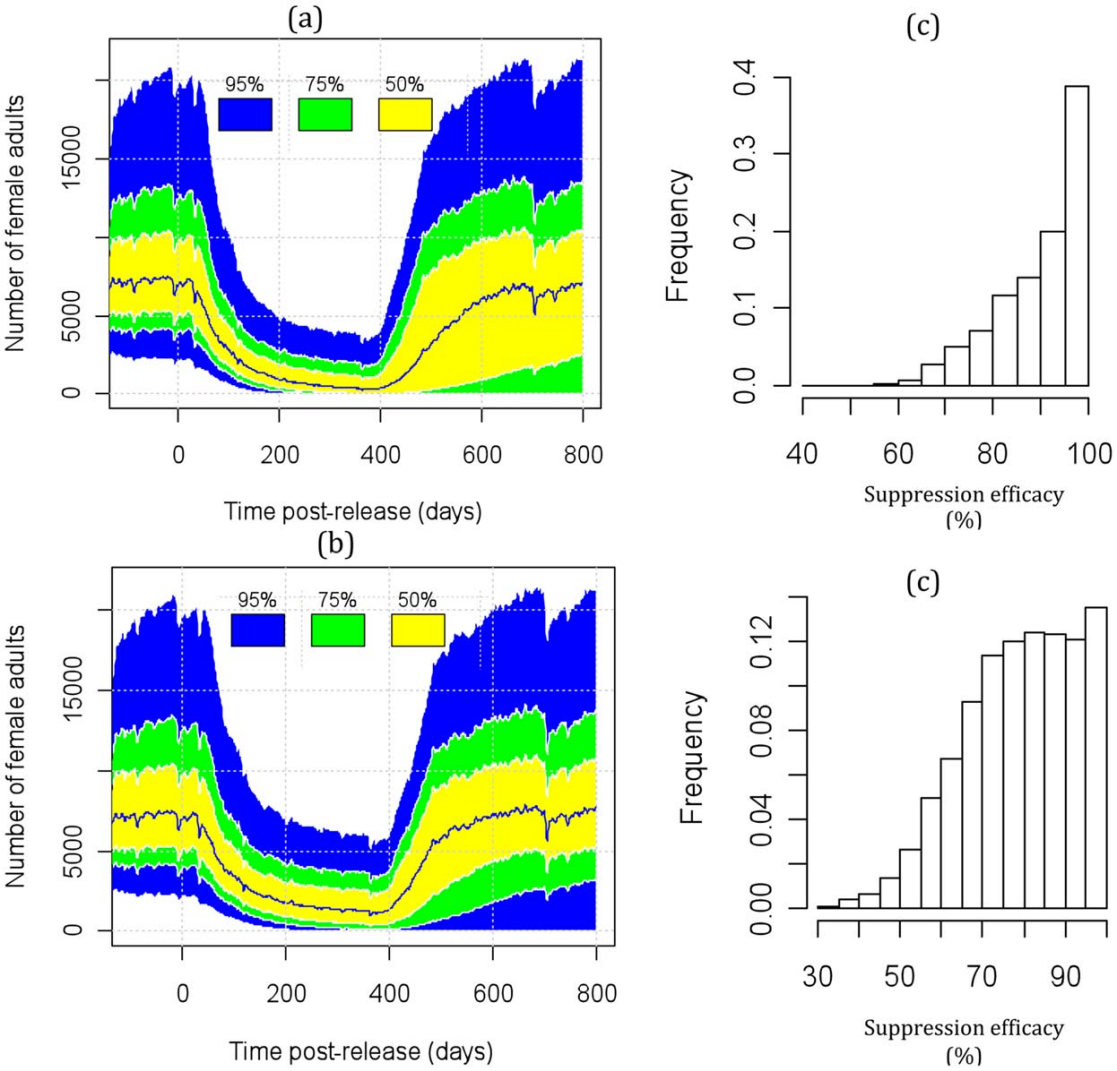
Figure 5. Uncertainty in the predicted population density across the entire simulated area with weekly releases of FK male adults for one year. Adults carrying one FK construct are released into the resident population (with natural heterogeneity). a) homogeneous male adult releases; b) point source uniform male adult releases. The yellow, green and blue shadings represent the 50%, 75% and 95% confidence intervals of the prediction, respectively. Grey represents the output boundary of all runs. The release starts at day 0 with burn in time of 200 days for the population to reach equilibrium. The suppression efficacy is measured by the percentage reduction in female adult population density, based on the reference density averaged over 3 weeks before the release and the suppressed density averaged over days 200–400 after the release. Histograms of mosquito population suppression efficacy in the 5000 model runs for release scenarios (a) and (b) are shown in panels (c) and (d).

**Figure 6:**
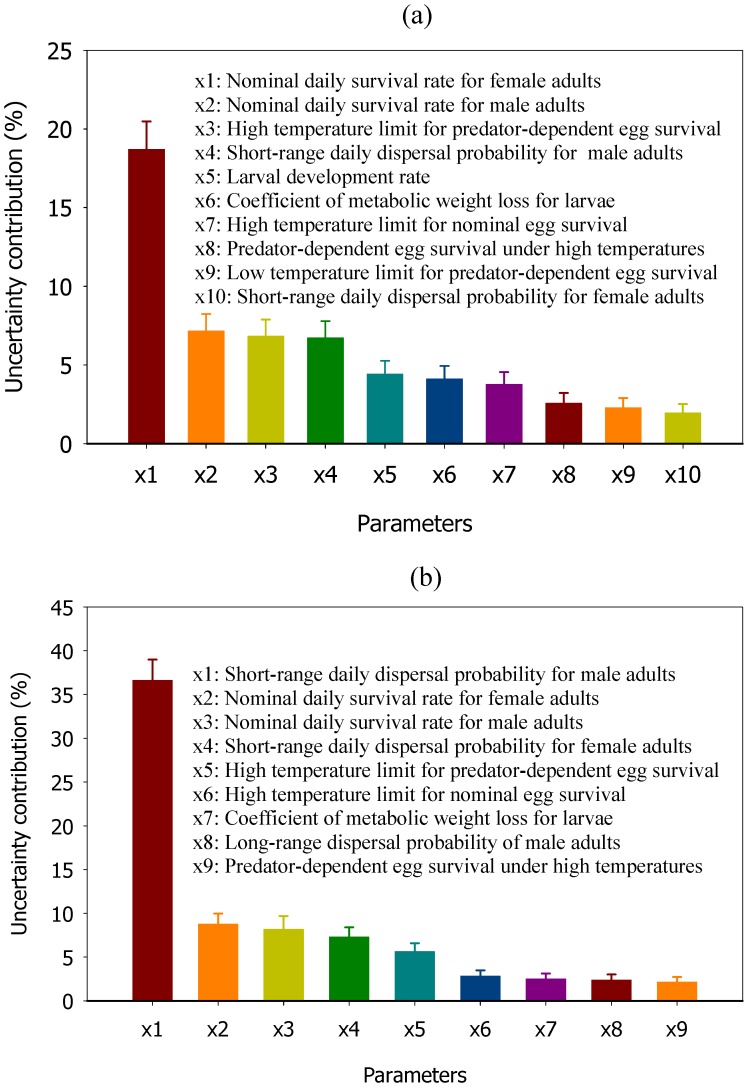
Figure 6. Uncertainty contributions by model parameters for the predicted suppression efficacy. Adults carrying one FK construct are released into the resident population (with natural heterogeneity). a) homogeneous male adult releases; b) point source uniform male adult releases. The suppression efficacy is measured by the percentage reduction in female adult population density, based on the reference density averaged over 3 weeks before the release and the suppressed density averaged over days 200–400 after the release. The vertical bars represent the 95% confidence intervals. To simplify the figures, we only plot those parameters that contribute more than one percent to the uncertainty.

We used uncertainty analysis to determine how inaccuracies inherent to our estimates of specific model parameters could affect model outcomes [34]. To suppress the confounding effect of initial release ratio (ratio of released male adults to wild male adults) on the importance of different model parameters, we set the ratio of released male adults to wild male adults to 2.5 in each of 5000 runs of the model with varied parameter values. The resulting population suppression after one year ranges from 55% to 100% for releases into each house, and from 35% to 100% percent for releases into 10% of houses (Fig. 5). The identified parameters that have important contributions to the model’s predictive uncertainty generally are those that have important impacts on mosquito reproduction number and larval population regulation (Fig. 6 and see Text S2 for further detail on these parameters). The rate of nominal adult female survival is the most important uncertainty source for homogeneous releases, contributing to about 20% of uncertainty in the predicted suppression efficacy (Fig. 6A), with higher female survival (i.e. longer generation time) leading to less mosquito population suppression in one year (Fig. S4A). On the other hand, larger values for FK male survival resulted in greater mosquito population suppression (Fig. S4B), presumably because long-lived FK males move further and mate more times. For point source releases, adult male dispersal is the largest uncertainty source, contributing about 35 percent of the total uncertainty in the predicted suppression after one year of releases (Fig. 6B). As expected, higher dispersal probability leads to a higher suppression efficacy (Figs. S4C and S5C). Notice that even for the homogenous release the adult male dispersal is an important parameter affecting mosquito suppression, explaining about 5 percent of uncertainty in the predicted suppression efficacy (Fig. S5A). This is presumably because the resident population is heterogeneous, and high density local subsets of households are effectively controlled not only by male adults released *in situ*, but also those released in nearby regions. Overall, greater male dispersal appears to reduce the effect of resident heterogeneity on population suppression (Fig. S4C).

## Discussion

Control strategies based on the release of engineered mosquitoes carrying conditional lethal genetic elements are conceptually feasible [12], [18], and the historical success of SIT approaches in eliminating populations of several pest species is encouraging [35]. There are, however, documented cases of unsuccessful SIT programs, particularly in the case of mosquitoes [4]. While those failures can be attributed to a number of biological and social factors, they teach us that there is an important gap between simple theoretical analyses and successful control in the field.

Several steps can be taken to overcome this gap, including the use of small-scale, open field releases. Although such trials are indeed helpful for initial assessments of feasibility and can be used for model calibration, it is impossible to extrapolate solely from such limited releases to the dynamics expected in larger urban areas. Hundreds of small releases would need to be carried out to account for stochastic variation, and sampling for a few remaining mosquitoes after a release is too logistically difficult to be certain that wild-type mosquitoes had been eliminated [36]. Biologically-detailed models can investigate the expected impact of known complexities arising in the field and therefore constitute a crucial tool for bridging the gaps between results of small scale experimental releases, simple models, and city-wide releases. While simple models of FK releases predict local elimination, this study illustrates that complex factors such as stochasticity, life history of the target species, spatial heterogeneity, and social compliance can result in very different predictions.

Even with the added realism in our model, there are limitations regarding the applicability of its predictions to large-scale implementations of this control strategy. First, while the additional level of detail accounts for some specific aspects of a natural mosquito population, no model can claim to capture the entire complexity of such a population. The gap between theoretical studies and field applications can therefore be narrowed, but remains present nonetheless. Second, complex models are generally less tractable than simpler models, and their predictions are subject to more uncertainty arising from the higher number of parameters and biological assumptions. This trade-off is an unavoidable cost of incorporating more realism into a model, and therefore requires more caution in interpreting the results. In the case of the Skeeter Buster model, the impact of the parametric uncertainties on model predictions was the subject of a previous, separate analysis [34], and we also provide in this study a specific uncertainty analysis of model predictions of FK control strategies outcome. Additionally, Skeeter Buster operates in a location-specific fashion that requires heavy parameterization to fit a particular location of interest. For this reason, this study was limited to one geographical and ecological setting, the city of Iquitos. The applicability of Skeeter Buster to this particular location has been investigated in a separate study [28], and allows us to proceed with more confidence towards investigating control strategies. It should be kept in mind, however, that while general patterns observed in these simulations can inform the development of control programs, specific predictions obtained in this setting cannot be directly ported to other, potentially very different locations without further, specific analysis.

With this framework in mind, the comparison of the various scenarios presented here provides important guidelines for the design of FK control strategies. First, our model predicts that complete elimination of an *Ae. aegypti* population from a large area such as a modern, urban city is an unrealistic objective, unless nearly homogeneous releases can be achieved for a long time period and the mosquito habitat is also relatively homogeneous. In those cases where our simulations indicate that elimination may be feasible, the possibility of *Aedes aegypti* reintroduction from neighboring populations, which was not considered in our simulations, will present an additional challenge for elimination, particularly in locations less isolated than the city of Iquitos that was the focus of this study. A sustained surveillance and control effort will therefore be required beyond the time frame predicted for local eradication. Elimination, however, is not necessarily the objective when controlling mosquito populations. Reducing the vector population below a certain threshold density is sufficient to prevent the local transmission of mosquito-borne disease [37]. The challenge with targeting a threshold density is that values for the threshold can be spatially and temporally dynamic based on underlying patterns of herd immunity in human populations. If this threshold is not reached, or not consistently reached (spatially and temporally), virus transmission could still occur (a question which the model used in this study cannot address). A successful strategy would therefore require a sophisticated system of epidemiologic and entomologic surveillance with the capacity to rapidly respond to elevated risk [38].

Second, if the release methodology is constrained to a percentage of households for practical purposes, the model predicts that, while being markedly less efficient than releases to each house, this approach can lead to substantial population suppression if release sites are distributed as evenly as possible. Since a perfectly regular distribution of sites in dengue-endemic cities will not be possible, the choice of release sites should be such that no region of the targeted area is too far removed from the closest release site. How far is too far depends on several traits of the FK males’ life history, including their dispersal ability (rate and distance) as well as their survival rates. Based on data in the literature [16], native adult males in our model had an average lifespan of 4.35 days and their daily movement was only to adjacent houses, reflecting the observation that *Ae. aegypti* adults do not move much among houses [39]–[41]. In most of the scenarios described the same assumptions about movement were ascribed to released, transgenic mosquitoes. Therefore, locations that are more than 5 houses away from release sites were unlikely (<1% probability) to be reached by any released FK male. A recent series of male-only releases outside of houses found that the majority of males had movement limited to a single block, but a small fraction moved more than 100m [32]. We ran the model for a uniform point source release with between 10%–50% of released males moving randomly to any site within the 2448 house area. Under these conditions, extinction was achievable with a substantially lower number of males than in scenarios with only local movement (Figure 4). In these scenarios we kept male mortality at default levels, but it is likely that males with greater movement encounter more mortality factors (e.g desiccation, predators). Further empirical studies are needed to examine these important parameters.

Third, our model provides a quantitative framework to compare the efficacy of releasing different life stages. Ultimately, the decision to release a given life stage has to include economic considerations, as it is likely that the costs per capita of rearing and releasing one adult or one egg would differ considerably. A potential benefit of releasing eggs is that the emergence of FK males from egg cohorts is spread over several days due to variation in larval development time to pupation [16], [42]. This would create several cohorts of FK males from a single release event that can disperse and mate with wild-type females emerging on several consecutive days in or around release sites. While weekly releases may be advantageous from an operational point of view, this strategy could offer an effective advantage in reducing the delay between releases, potentially improving the efficacy of population suppression [43].

Finally, model output is consistent with predictions that strains with several unlinked lethal genetic constructs lacking fitness costs for males could increase the efficacy of population suppression [18], defined either as the probability of complete elimination or as the time needed to reach a 90% reduction (see Fig. S6 for confirmation with up to four independent elements). When the number of independent elements is increased, the second-generation offspring (and generations thereafter) of an FK adult are more likely to inherit at least one lethal transgenic element, thus perpetuating more efficiently the population suppression effect of a single release. It should be noted, however, that when fitness costs exist, the inclusion of additional elements could be detrimental [18] (and see Fig. S1).

Our simulations indicate that spatial heterogeneity in local mosquito densities will make area-wide elimination, or any given level of population suppression, more difficult to achieve than if the population is homogeneous. With homogeneous release patterns across heterogeneous mosquito populations like those that we considered, local FK to wild-type ratios will vary across the simulated area, with insufficient suppression efficacy in regions with high mosquito production. This issue could be resolved by raising the release ratios across the entire area to accommodate for the highest producing regions, or by moving away from homogeneous release patterns to locally adjusting release levels based on mosquito abundance, to the extent that it can be characterized with confidence. This decision would be primarily guided by cost-effectiveness analyses. The quantitative assessments provided by model-based studies such as ours constitute a crucial piece of that assessment.

It appears likely that genetic pest suppression programs like the ones considered in this study will have to be maintained over a long period of time. This is apparent in our simulations in those cases where local elimination proves unfeasible, but long term management will also be required in conditions more amenable to local eradication. In all cases, the possibility of reintroduction from outside populations, either through natural mosquito migration or through longer range, human-mediated dispersal, will present a threat of re-establishment if the program is discontinued. This makes it critical to assess the sustainability of population suppression efforts especially when there is high reliance on community involvement. Bottom-up approaches are favored in community-based health control programs [27], [44], and their potential is illustrated by the successful biological control of dengue vectors by *Mesocyclops* predators distributed to local households in Vietnam [45]–[47]. These approaches, however, present significant and still poorly quantified challenges to long-term sustainability [21], [33], [48]. We emphasize here that it is important to ensure that targeted coverage is maintained across the treated area throughout the release program, including when population levels have been reduced below easily detectable levels. The different delivery methods we have considered in this analysis (delivery of eggs or adults, from community-based release sites or through a more centralized system via aerial or ground-based delivery systems) will likely face different challenges with regards to this sustainability. Strategies that rely heavily on community involvement (e.g. distribution of dried eggs that have to be hatched on a regular basis) offer great coverage possibilities, but are likely more prone to a decrease in participation as the program goes on, compared to more centralized and globally managed methods. These considerations, based on social, economical and political factors that are beyond the scope of this study, will nevertheless have to be accounted for in operational decisions. Because elimination is unlikely, the mosquito population could quickly recover in uncontrolled areas if coverage was dropped after a period of population reduction.

Our model predictions, like those of other models, are affected by a number of sources of uncertainty. Our uncertainty analysis identified specific parameters that need to be more carefully measured to achieve more robust model-based guidance of *Ae. aegypti* operational control implementations. Longer development times at different stages contribute to lower suppression efficacy, suggesting that a desired level of suppression might take longer to achieve in areas with lower food inputs and, thus, longer larval development time. Adult dispersal is understandably an important factor when releases are carried out only at selected sites, but is still important in homogeneous release scenarios because it helps alleviate the effects of spatial heterogeneity. The precise pattern of *Ae. aegypti* dispersal is likely to vary between ecologically different locations [41], and the dispersal algorithm included in Skeeter Buster constitutes only one example of such a pattern. More empirical detailed data on these parameters would therefore be needed to reduce major sources of uncertainties and enable implementation of more efficient FK control programs. Additionally, while this study focuses on the impact of FK strategies on the vector population, the resulting effect on dengue transmission is often non trivial, as it depends not only on the level of population reduction that can be achieved, but also, among other factors, on the heterogeneity in the remaining vector population and on possible changes in the age structure of this population. The epidemiological impact of such control programs should therefore be the subject of further investigation.

Our analysis highlights the utility of detailed models for addressing questions that public health officials, regulatory bodies, and funding agencies are asking about the feasibility and risks associated with genetic strategies for prevention of mosquito-borne disease. Similar insights are not obtainable from relatively simple models that do not account for key heterogeneities and are beyond the logistic and financial resources of empirical approaches alone. In combination with progressively detailed ecological and economical investigations, complex simulation models like ours provide valuable insights into otherwise difficult to tackle, precise comparisons between the feasibility and desirability of different spatially- and temporally-explicit genetic release strategies.

## Methods

### Model Description

In this study we used the Skeeter Buster model [16], a stochastic, biologically detailed, spatially-explicit model of *Ae. aegypti* populations, based on biological elements of the CIMSiM model [49]. Because of the complexity of the model, we do not detail all of its features here. Instead we provide a succinct description of model components critical to understanding this study. For further description of these specific components, see Text S2. For a complete description of the model, and the empirical data upon which it is based, see reference [16].

The Skeeter Buster model simulates the biological development of four life stages of *Ae. aegypti*: eggs, larvae, pupae in individual water-holding containers, and adults in individual houses. Houses are laid out on a rectangular grid, and containers are assigned to their specific house on the grid. The model includes a detailed description of temperature-dependent development rates of all life stages. Density-dependent effects of intraspecific competition in the larval stages are described using the model by Gilpin and McClelland [50] that tracks the parallel changes in food amounts in each breeding site and larval weight. Larval weight (and potential starving) in turn affects survival and development time. Adult females are assumed to be strictly monogamous [51] and for each unmated female, a mate is selected at random (with a probability proportional to its weight) among all males present in the same house; if no males are present, that female will remain unmated until the next day. Adults can move from one house to one of its immediate neighbors with a daily probability of 30%, a value obtained from field dispersal studies (see reference (32)). This probability is identical for adult males and females. We assume that humans are present in all simulated premises, and consequently that the dispersal probability is not affected by the status of the female (host-seeking or resting). The direction of this short range dispersal is chosen at random. Therefore, while daily dispersal is limited to nearest neighbors, the potential lifetime dispersal distance is much larger. The model also contains a function for long-distance movement, and this was used to simulate the initial movement of released males.

### Study Area

We choose to simulate mosquito populations in a part of the city of Iquitos, Peru, because the application of Skeeter Buster to this location has been the object of a specific case study [28]. Iquitos is located in the northeast portion of the Amazon Basin of Peru (location: 73.2W, 3.7S) and has a tropical climate with no marked seasonality. The city is relatively isolated, with no road connection to other urban centers in Peru or in neighboring countries. Detailed descriptions of the study area and its *Ae. aegypti* population are available from earlier published studies [30], [31]. We simulate a 2448-house area (68×36) parameterized according to data collected in a heavily surveyed region of the city [28].

### Simulating FK Strain Release in Skeeter Buster

FK was simulated in Skeeter Buster as a female-specific lethal allele carried on a single locus with two alleles. When strains with several insertions of the same FK construct were considered, the insertions were assumed to occur on separate linkage groups, and, therefore, segregate independently. Lethality occurred on the first day that an adult female with one or more copies of the FK allele emerges from pupation. These conditions match the characteristics of the FK strain of *Ae. aegypti* developed by Fu et al. [17] in which emerging females cannot fly and are effectively removed from the population at emergence due to their inability to locate mates or take blood meals.

Each run of the model was initiated with 20 eggs in every container, and there was a 1-year burn-in period before releases to allow the mosquito population dynamics to stabilize. For each scenario the model was run 30 times.

### Release Scenarios

The release of adults was modeled by adding cohorts of the same number of homozygous FK males to each desired grid location (i.e. individual premises) in the model space. The same locations were used for each weekly release. To simulate release of eggs, following the general scenario put forward by Fu et al. [17], additional containers were incorporated into the model every week in the corresponding release houses. These containers had an initial supply of nutritional resources that ensures favorable larval development of the released eggs, but were shielded from ovipositing females. An identical number of male and female eggs were input into the container, however, because we consider only the case of 100% efficiency of the female-specific lethal elements, only male adults emerged from these containers. They were then removed from the simulated area as soon as all released individuals had either died or emerged as adults.

We simulated three distinct spatial release approaches to examine the impact of dispersion of the transgenic mosquitoes on outcomes. The spatial patterns we consider represent ideal release scenarios, and while they might be operationally impossible to replicate exactly, comparison between them is informative as to what actual release programs should aim for. Homogeneous releases consisted of releasing the same number of transgenic mosquitoes in every house in the simulated area. Because coverage approaching this pattern appears achievable only through aerial coverage of a city, we only applied this spatial pattern to adult releases. Point source releases corresponded to selection of a discrete subset of 10% of houses as release sites. We define two distinct patterns of point source releases depending on the process of selection of these release sites: random, in which 242 release sites were randomly chosen among the 2448 simulated houses and uniform in which 242 sites were laid out at regular intervals on the grid. In both cases, release sites remained fixed throughout the release period.

### Early Male Adult Dispersal

When a large number of adult males are simultaneously released in the same site, we simulate scenarios where only a (deterministically set) fraction of these released males will remain in the release site on the day of release. Each remaining male in the released cohort is then immediately allocated to another house in the simulated area, chosen at random (and is therefore never assigned to the release site). We consider cases where 0%, 10%, 20% or 50% of the released cohort are such early dispersers (and therefore only 100%, 90%, 80% or 50%, respectively, of the released mosquitoes are placed upon release in the designated release site).

### Heterogeneity

We simulated releases in Iquitos, where the spatial heterogeneity in mosquito production among houses is relatively limited [30], [31], [52]. In more heterogeneous environments (e.g. Tapachula, Mexico [53], [54]), however, spatially homogeneous mosquito releases could result in heterogeneous ratios of transgenic to local mosquitoes among houses [55]. We model situations where we artificially increased the variation in pupal productivity among houses by transforming each house into either a high-producing house (with probability 1/*Φ*, where *Φ*≥2) or into a low-producing house (with probability 1−1/*Φ*). In the former case, every container found in that house was included *Φ*−1 times in the corresponding house in the simulation, while in the latter case, every container found in that house had a probability 1/(*Φ*−1) to be kept in the simulation, and is otherwise removed. With this method the average total number of containers in the city remained identical to the default situation. The case 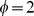 leaves the original distribution unchanged, while integers values of 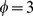 or larger increase the heterogeneity in container distribution among houses. Applied to a perfectly homogeneous setting, this method would result in a coefficient of variation in container distribution among houses equal to 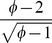, i.e. 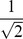 or approx. 71% for 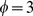, and 150% for 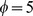.

### Pre-release Population Reduction

We simulated pre-release control programs based on traditional methods, consisting of adulticidal control applied periodically in premises across the modeled area. Two weeks of insecticide application were simulated, causing an assumed 90% additional mortality among adults every day, ending on the day before the onset of FK releases. Throughout that period, we considered that each premise had a 10% probability of not receiving insecticide on any given day, based on the assumption that coverage cannot reach every premise in the targeted urban area. Supplementary runs were run with an increased (20%) probability of not receiving insecticide (Figure S7), showing little to no difference compared to the default value.

Additional methods (community participation and uncertainty analysis) are presented in Text S1.

## Supporting Information

Figure S1 **FK elements with a fitness cost.** Proportion of simulations that reach extinction after 3 simulated years when adult males are released weekly in every house (homogeneous strategy) when FK elements are associated with a fitness cost. Fitness cost is defined as *c* so that the relative fitnesses of wild-type, heterozygous and homozygous at any FK locus are 1, (1−*c*)^0.5^ and (1−*c*) respectively. When multiple loci are involved, fitness is calculated multiplicatively across loci. Circles: *c* = 0 (from Fig. 1). Squares (dashed line): *c* = 0.25. Diamonds (dotted line): *c* = 0.5. Gray symbols: one FK element. Black symbols: three independent FK elements. Note the stronger effect of costs on strains carrying three FK elements.
(TIF)

Figure S2 **Time to 90% reduction with increased resident heterogeneity.** Time to reach 90% population reduction when male adults carrying 1 lethal elements are released in every house (homogeneous strategy) into a population with increased heterogeneity in container distribution. For each scenario 30 replicated simulations are run. Circles: unchanged distribution *Φ* = 1; diamonds: increased heterogeneity *Φ* = 3; squares: increased heterogeneity *Φ* = 5. Error bars represent standard deviation.
(TIF)

Figure S3 **Effects of decreasing community participation.** Time series of number of adult females in the population with decreased compliance rates to FK point source releases of eggs (100, 300 or 500 eggs per week and per site). Compliance rate decreases weekly, at a rate equivalent to a 30% decrease in compliance per year. Solid line: average of 10 simulations. Dashed lines: minimum and maximum. A: uniform distribution of release sites. B: random distribution.
(TIF)

Figure S4 **Dependence of mosquito population suppression efficacy (%) on model parameters for homogeneous release of male adults.** Adults carrying one FK construct are released into the resident population (with natural heterogeneity). The suppression efficacy is measured by the percentage reduction in female adult population density, based on the reference density averaged over 3 weeks before the release and the suppressed density averaged over days 200–400 after the release. The lines are fitted to the scatterplot of parameter values sampled by FAST and the corresponding population density using a cubic smoothing splines with the SemiPar R package (Wand et al., 2005). The shaded areas are the 95% confidence intervals of the fitted lines.
(TIF)

Figure S5 **Dependence of mosquito population suppression efficacy (%) on model parameters for point source male adult releases within 10% of the houses (uniformly distributed).** Adults carrying one FK construct are released into the resident population (with natural heterogeneity). The suppression efficacy is measured by the percentage reduction in female adult population density, based on the reference density averaged over 3 weeks before the release and the suppressed density averaged over days 200–400 after the release. The lines are fitted to the scatterplot of parameter values sampled by FAST and the corresponding population density using a cubic smoothing splines with the SemiPar R package (Wand et al., 2005). The shaded areas are the 95% confidence intervals of the fitted lines.
(TIF)

Figure S6 **Time to 90% population reduction with releases of eggs in 10% of uniformly selected release sites.** For each scenario 10 replicated simulations are run (average ± SD shown). Open symbols: release of individuals carrying two FK elements. Filled symbols: release of individuals carrying four FK elements. FK adults are released into the resident population with natural heterogeneity.
(TIF)

Figure S7 **Impact of coverage during pre-release control.** Proportion of simulations that reach extinction after 3 simulated years when males are released weekly in every house. Filled symbols: 90% coverage of pre-release control. Open symbols: 80% coverage. Gray lines: 1 lethal element (1 K). Black lines: 3 lethal elements (3 K). Error bars show estimated proportion +/− standard error (calculated as in Figure 1).
(TIF)

Text S1 **Supplementary methods.**
(DOC)

Text S2 **Supplemental details of the Skeeter Buster model.**
(DOC)
