# Supplementary figures and images for: Assessing the Feasibility of Controlling Aedes aegypti with Transgenic Methods: A Model-Based Evaluation
Source: PLoS One. 2012 Dec 21;7(12):e52235. doi: 10.1371/journal.pone.0052235 (PMC3528761; doi:10.1371/journal.pone.0052235)

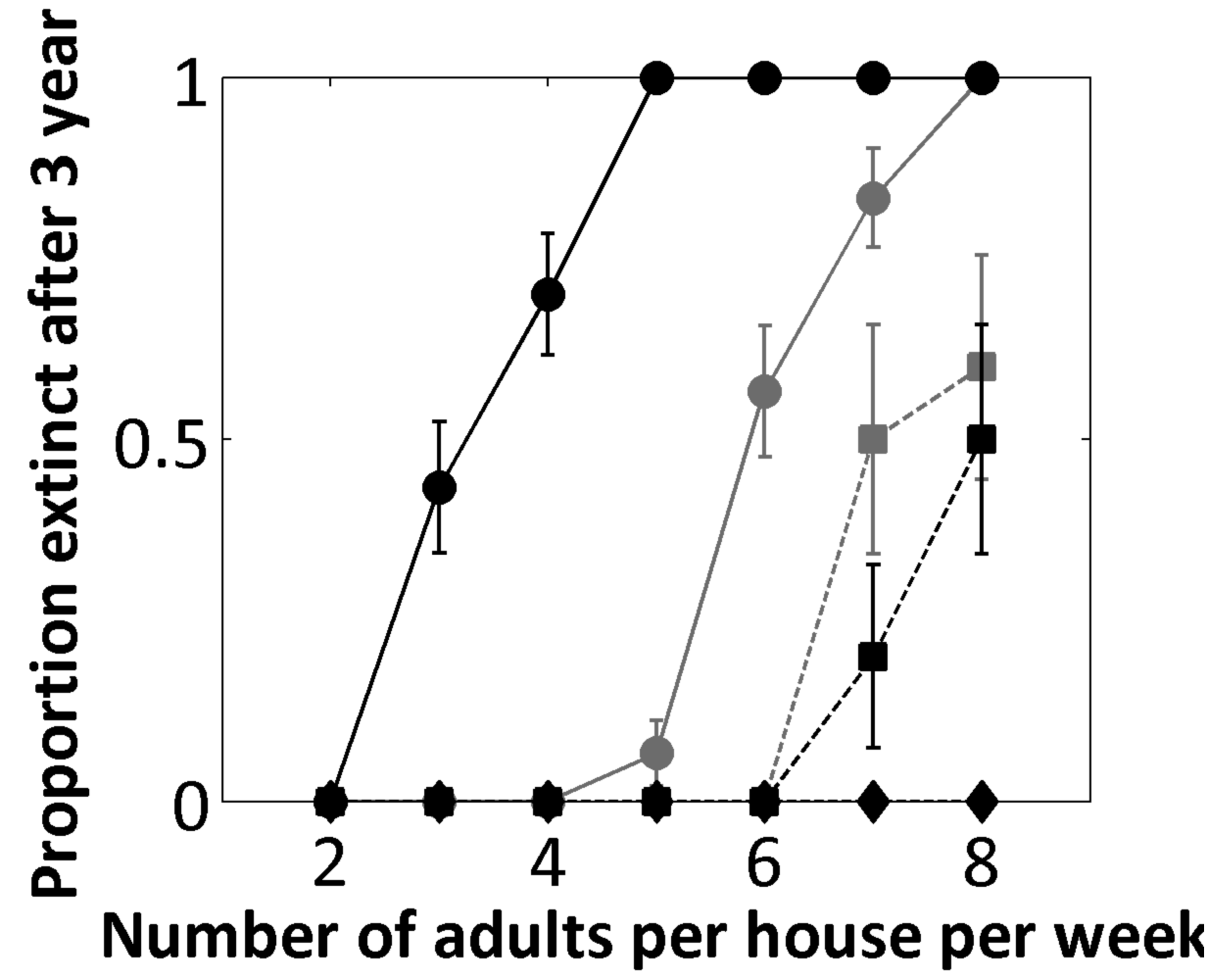

Supplement: Figure S1 — FK elements with a fitness cost. Proportion of simulations that reach extinction after 3 simulated years when adult males are released weekly in every house (homogeneous strategy) when FK elements are associated with a fitness cost. Fitness cost is defined as c so that the relative fitnesses of wild-type, heterozygous and homozygous at any FK locus are 1, (1−c)0.5 and (1−c) respectively. When multiple loci are involved, fitness is calculated multiplicatively across loci. Circles: c = 0 (from Fig. 1). Squares (dashed line): c = 0.25. Diamonds (dotted line): c = 0.5. Gray symbols: one FK element. Black symbols: three independent FK elements. Note the stronger effect of costs on strains carrying three FK elements. (TIF) [file pone.0052235.s001.tif]

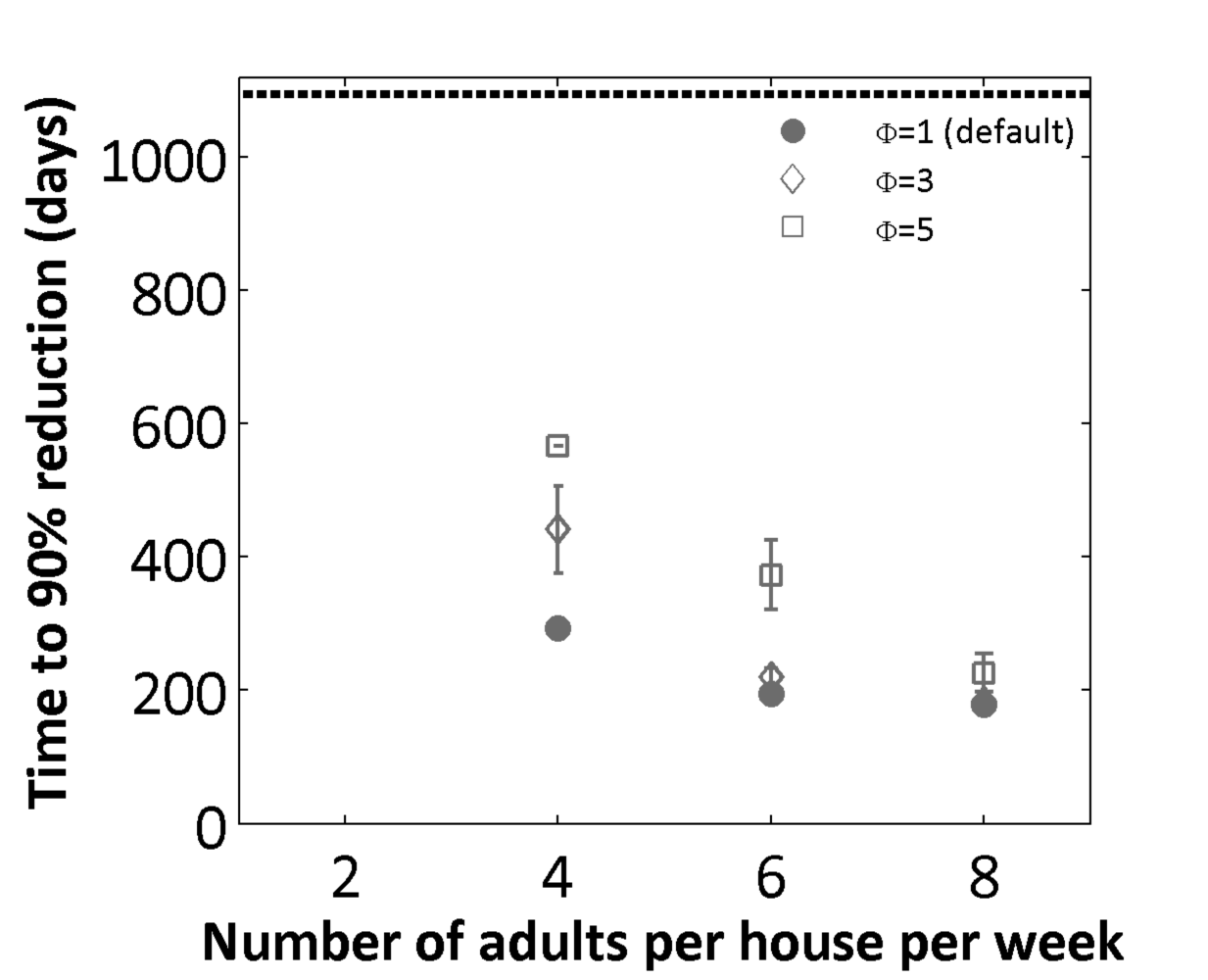

Supplement: Figure S2 — Time to 90% reduction with increased resident heterogeneity. Time to reach 90% population reduction when male adults carrying 1 lethal elements are released in every house (homogeneous strategy) into a population with increased heterogeneity in container distribution. For each scenario 30 replicated simulations are run. Circles: unchanged distribution Φ = 1; diamonds: increased heterogeneity Φ = 3; squares: increased heterogeneity Φ = 5. Error bars represent standard deviation. (TIF) [file pone.0052235.s002.tif]

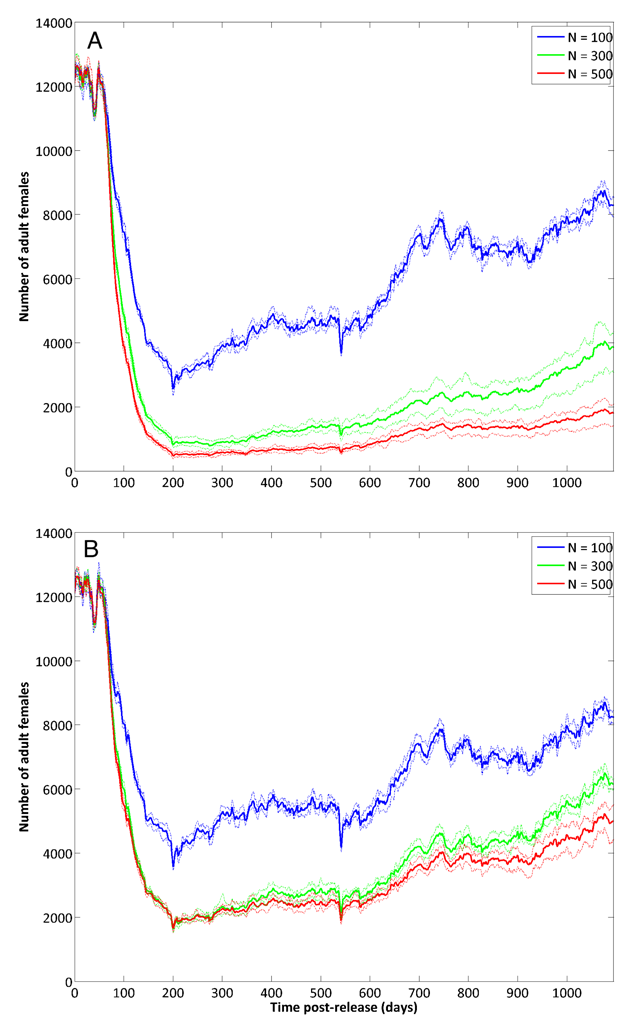

Supplement: Figure S3 — Effects of decreasing community participation. Time series of number of adult females in the population with decreased compliance rates to FK point source releases of eggs (100, 300 or 500 eggs per week and per site). Compliance rate decreases weekly, at a rate equivalent to a 30% decrease in compliance per year. Solid line: average of 10 simulations. Dashed lines: minimum and maximum. A: uniform distribution of release sites. B: random distribution. (TIF) [file pone.0052235.s003.tif]

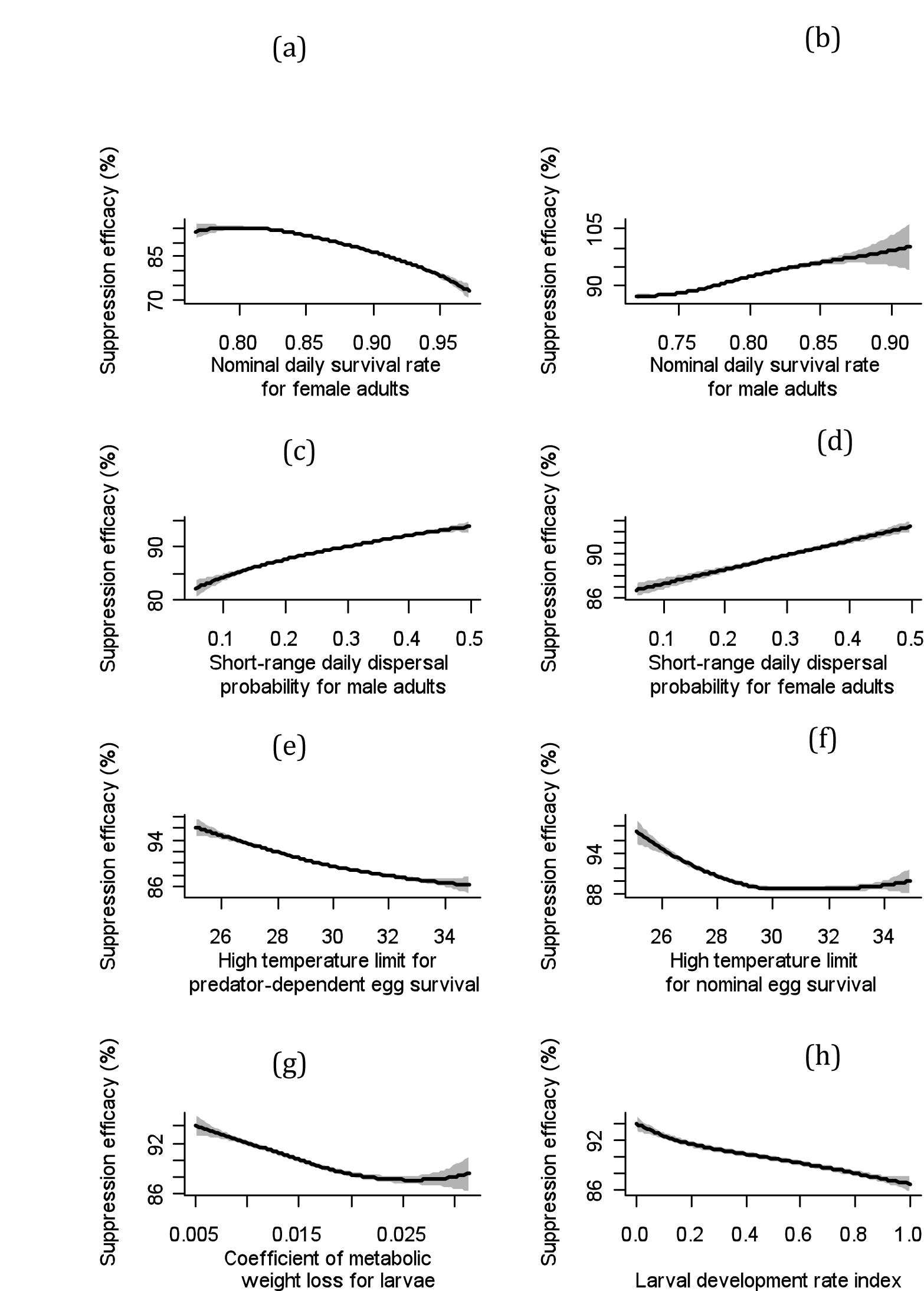

Supplement: Figure S4 — Dependence of mosquito population suppression efficacy (%) on model parameters for homogeneous release of male adults. Adults carrying one FK construct are released into the resident population (with natural heterogeneity). The suppression efficacy is measured by the percentage reduction in female adult population density, based on the reference density averaged over 3 weeks before the release and the suppressed density averaged over days 200–400 after the release. The lines are fitted to the scatterplot of parameter values sampled by FAST and the corresponding population density using a cubic smoothing splines with the SemiPar R package (Wand et al., 2005). The shaded areas are the 95% confidence intervals of the fitted lines. (TIF) [file pone.0052235.s004.tif]

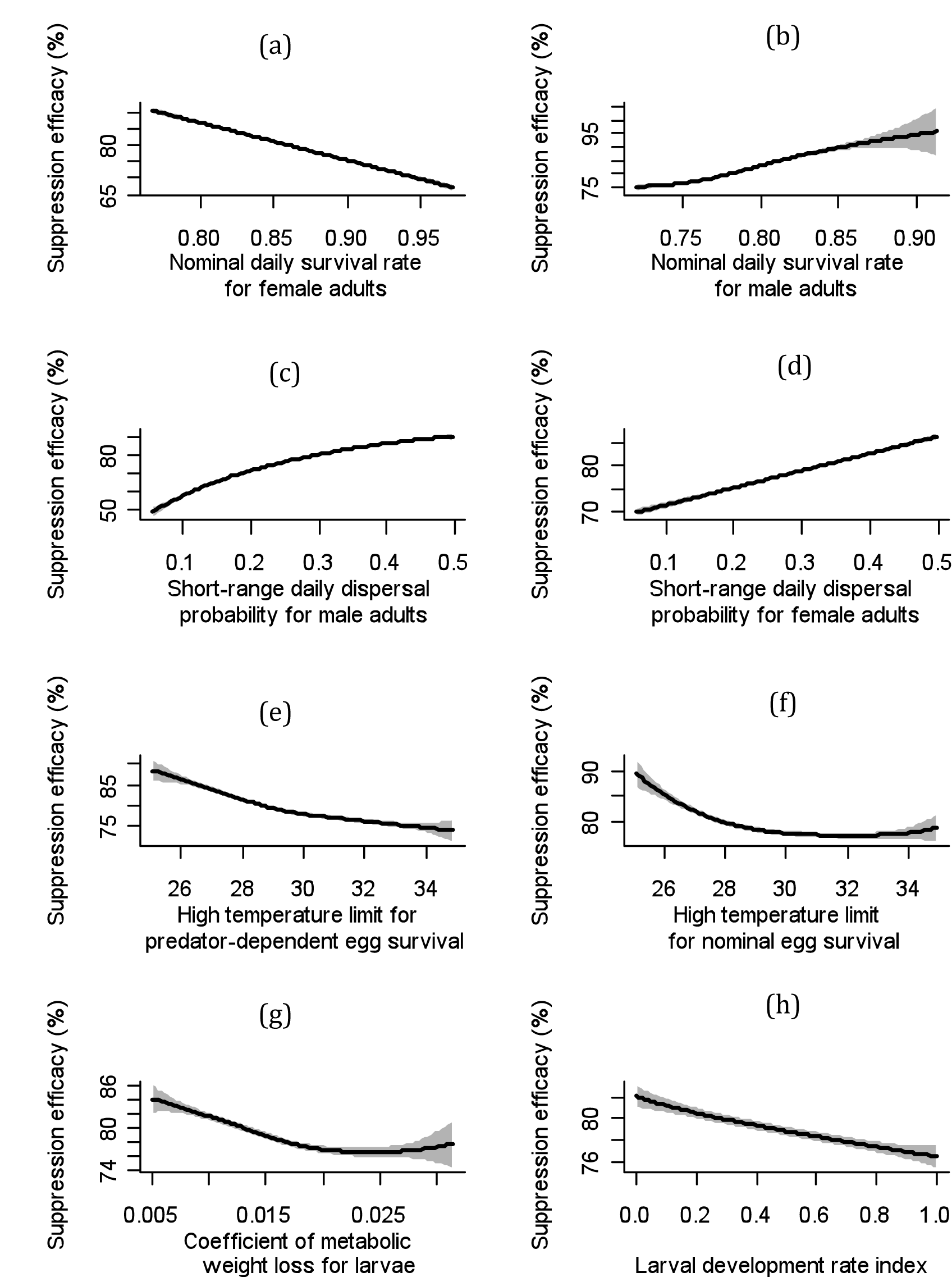

Supplement: Figure S5 — Dependence of mosquito population suppression efficacy (%) on model parameters for point source male adult releases within 10% of the houses (uniformly distributed). Adults carrying one FK construct are released into the resident population (with natural heterogeneity). The suppression efficacy is measured by the percentage reduction in female adult population density, based on the reference density averaged over 3 weeks before the release and the suppressed density averaged over days 200–400 after the release. The lines are fitted to the scatterplot of parameter values sampled by FAST and the corresponding population density using a cubic smoothing splines with the SemiPar R package (Wand et al., 2005). The shaded areas are the 95% confidence intervals of the fitted lines. (TIF) [file pone.0052235.s005.tif]

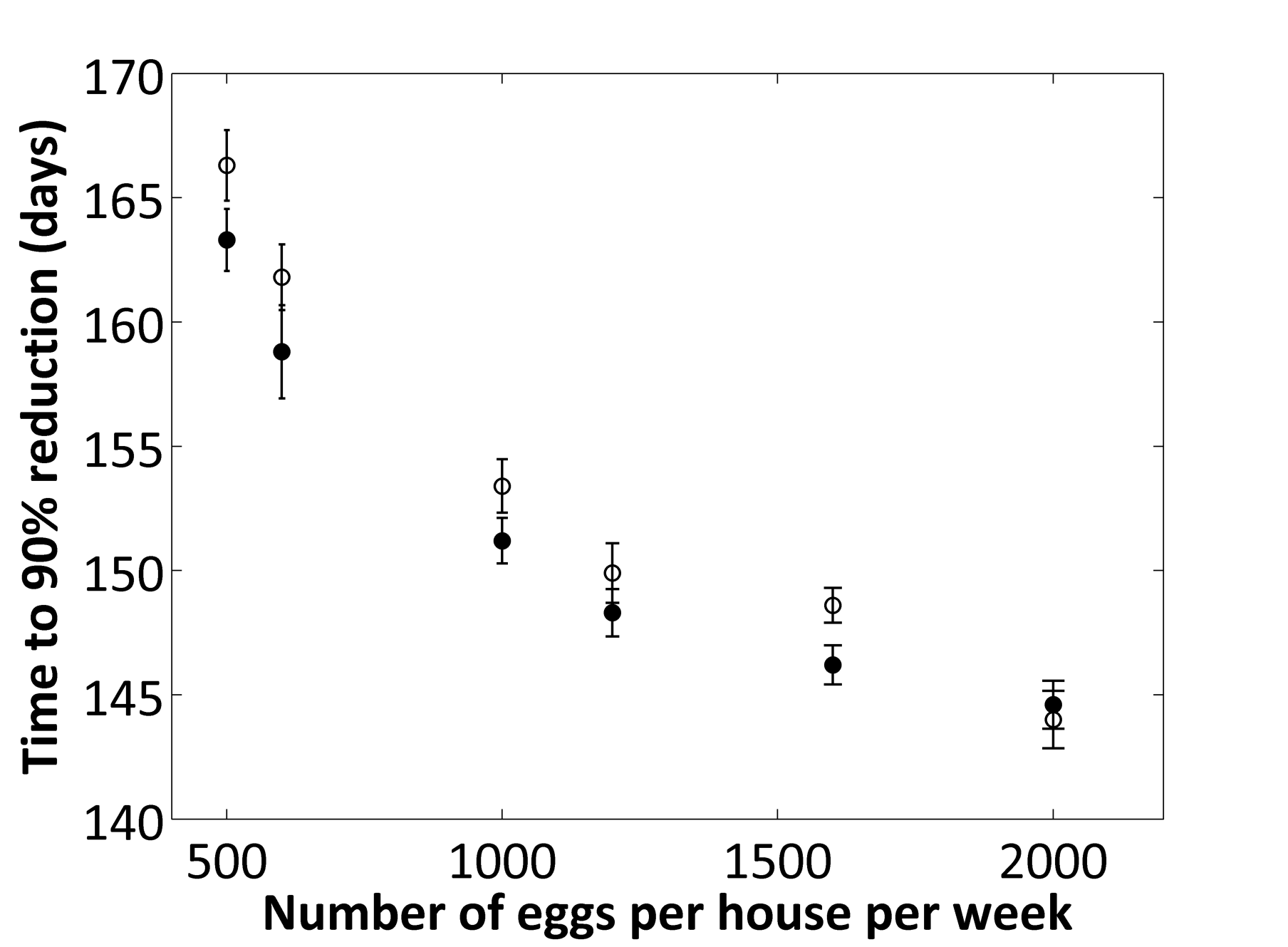

Supplement: Figure S6 — Time to 90% population reduction with releases of eggs in 10% of uniformly selected release sites. For each scenario 10 replicated simulations are run (average ± SD shown). Open symbols: release of individuals carrying two FK elements. Filled symbols: release of individuals carrying four FK elements. FK adults are released into the resident population with natural heterogeneity. (TIF) [file pone.0052235.s006.tif]

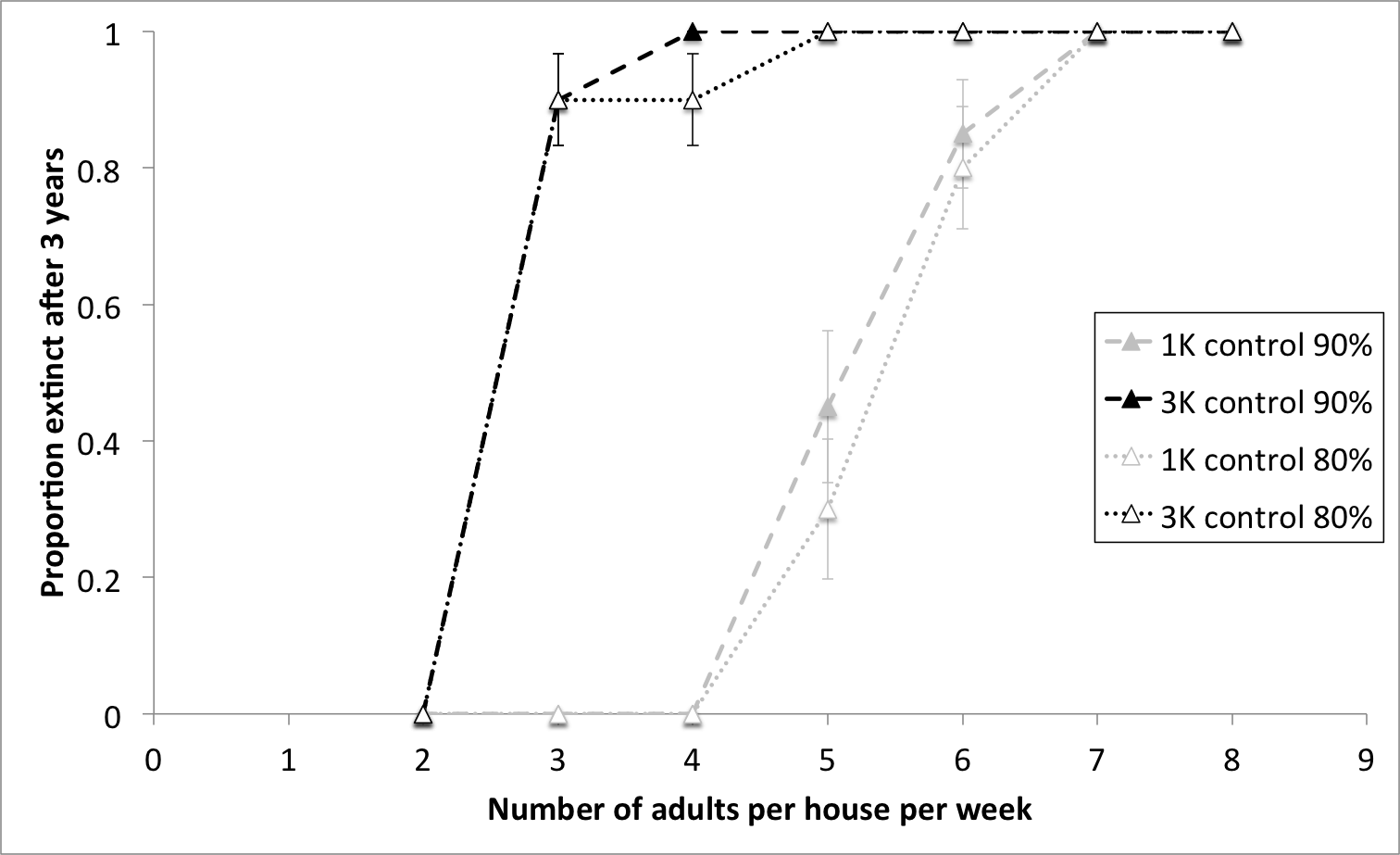

Supplement: Figure S7 — Impact of coverage during pre-release control. Proportion of simulations that reach extinction after 3 simulated years when males are released weekly in every house. Filled symbols: 90% coverage of pre-release control. Open symbols: 80% coverage. Gray lines: 1 lethal element (1 K). Black lines: 3 lethal elements (3 K). Error bars show estimated proportion +/− standard error (calculated as in Figure 1). (TIF) [file pone.0052235.s007.tif]
