# Supplementary material for: Assessing the Feasibility of Controlling Aedes aegypti with Transgenic Methods: A Model-Based Evaluation
Source: PLoS One. 2012 Dec 21;7(12):e52235. doi: 10.1371/journal.pone.0052235 (PMC3528761; doi:10.1371/journal.pone.0052235)
Supplement: Text S1 — Supplementary methods. (DOC) [file pone.0052235.s008.doc]

**Text S1**

**Supplementary Methods**

**Participation**

In scenarios where eggs were released and the local community or local officials were involved in the releases, changes in the level of participation with release operations can be expected. To that end, we considered that each release site had a given probability of dropping out of the program each week. Once a site dropped out, no release occurred from that site for the rest of the program. For convenience probabilities are shown in the text and figures as probabilities per year.

## Uncertainty analysis

Our uncertainty analysis examined uncertainties in model predictions resulting from parametric uncertainty (i.e., uncertainties in the estimates of 67 parameters that describe mosquito biology and life history) to identify key factors that affect our predictions of population suppression. We followed the method described in a previous uncertainty analysis of Skeeter Buster predictions .

Our analysis is based on the Fourier Amplitude Sensitivity Test (FAST) technique , which uses a periodic sampling of parameter space and a Fourier transformation to decompose the total variance of a model output into partial variances contributed by model parameters. In this study, we used an improved version of FAST developed by Xu and Gertner , which does not require an unfeasibly large sample size for complex models and can take correlations among parameters into account. We used the UASA ToolBox developed by Xu et al. . The standard errors of uncertainty contributions (i.e., the proportion of uncertainty in model prediction contributed by specific model parameters) were estimated using a delta method based on a single sample of size 5000 . The sample of size 5000 gave us reasonable precision (i.e., small standard errors) for the estimated uncertainty contributions .

We ran the FAST analysis for two release scenarios based on the release of male adults: homogeneous and uniform point source releases. For each scenario, we ran 5000 simulations based on parameter values sampled by FAST. Because the combination of parameter values in each simulation resulted in varied baseline (pre-release) levels of population density, we equalized the initial ratio of wild mosquitoes to released mosquitoes (ratio = 2.5: 1) for the 5000 simulations for fair comparison across simulations. In the analysis, we specifically examined the percentage reduction in female adult population density, based on the reference density averaged over 3 weeks before the release and the suppressed density averaged over days 200-400 after the release.

**References**
