# Supplementary material for: Assessing the Feasibility of Controlling Aedes aegypti with Transgenic Methods: A Model-Based Evaluation
Source: PLoS One. 2012 Dec 21;7(12):e52235. doi: 10.1371/journal.pone.0052235 (PMC3528761; doi:10.1371/journal.pone.0052235)
Supplement: Text S2 — Supplemental details of the Skeeter Buster model. (DOC) [file pone.0052235.s009.doc]

**Text S2**

**Details of the Skeeter Buster model**

**Nominal daily survival rates**

Skeeter Buster assumes that survival at all stages is independent of age and density (except for indirect larval fasting effects). Nominal daily survival probabilities are : 0.99 for eggs, larvae and pupae, 0.89 for female adults and 0.77 for male adults.

These survival probabilities can be modified by the multiplication of factors reflecting additional sources of mortality, notably temperature, as described in the following section.

**Temperature dependent survival probabilities**

For all stages, daily survival probability is multiplied by an additional factor *s_T_* reflecting temperature effects on survival. The value of this factor depends on the daily extreme temperatures *T*_min_ and *T*_max_, and on 4 stage-specific threshold temperatures. We can write:

*s_T_ = s_T_*_min_ ** s_T_*_max_

where:

- if *T*_min_ < *T*_0_ then *s_T_*_min_ = 0.05
- if *T*_0_ < *T*_min_ < *T*_1_ then *s_T_*_min_ = 0.05 + 0.95(*T*_min_-*T*_0_)/(*T*_1_-*T*_0_) (in other words, survival increases linearly with *T*_min_ from 0.05 at *T*_0_ to 1.0 at *T*_1_)
- otherwise, *s_T_*_min_ = 1.0 (no effect of minimum temperature on survival)

and:

- if *T*_2_ < *T*_max_ <*T_3_* then *s_T_*_max_ = 1 - 0.95(*T*_max_-*T*_2_)/(*T*_3_-*T*_2_) (in other words, survival decreases linearly with *T*_max_ from 1.0 at *T*_2_ to 0.05 at *T*_3_)
- if *T*_max_ > *T*_3_ then *s_T_*_max_ = 0.05
- otherwise, *s_T_*_max_ = 1.0 (no effect of maximum temperature on survival)

Values of the threshold temperatures for these four temperature-dependent survival factors are given in Table S1. Note that the temperatures used in the calculation of these factors are water temperatures for eggs, larvae and pupae, and air temperatures for adults.

**Table S1. Thresholds for temperature-dependent survival calculations (ºC)**

|  | ***Eggs*** | ***Larvae/Pupae*** | **Adults** |
| --- | --- | --- | --- |
| *T*_0_ | -14 | 5 | 0 |
| *T*_1_ | -6 | 10 | 4 |
| *T*_2_ | 30 | 39 | 40 |
| *T*_3_ | 47 | 44 | 50 |

Additionally, egg cohorts are subject to an additional source of mortality: predation. The activity of predators is itself assumed to be temperature-dependent in the following fashion:

- below a threshold temperature *T_0_* (“low temperature limit”, default 20ºC) no additional mortality is assumed,
- above a threshold temperature *T_1_* (“high temperature limit”, default 30ºC) a daily mortality probability of 30% is assumed,
- between *T_0_* and *T_1_* the mortality probability increases linearly between 0% and 30%.

**Larval growth and weight change**

Larval weight change and the associated changes in the amount of food in the container are modeled in parallel according to the following equations :

$$\frac{dW(t)}{dt}=af(T_{t})\left(W(t)^{b}\left(1-e^{-cF(t)}-d_{1}W(t)^{d_{2}}\right)\right)$$

$\frac{dF(t)}{dt}=-n(t)f(T_{t})W(t)^{b}\left(1-e^{-cF(t)}\right)$,

where *t* is time, *W*(*t*) is larval dry weight (mg), *F*(*t*) is the amount of food within the container (mg), *T_t_* is the temperature (K) at time *t* and *n*(*t*) is the number of larvae in the cohort. Parameter values and descriptions are detailed in Table S2

**Table S2. Parameter description and default values for larval weight and food amount calculations**.

| ***Parameter*** | ***Description*** | **Value** |
| --- | --- | --- |
| ***a*** | Conversion rate of consumed food to biomass | 0.3 |
| ***b*** | Exponent of increase of food exploitation rate with body weight | 0.8 |
| ***c*** | Change in food exploitation rate with food density (type II functional response) | 0.1 |
| ***d_1_*** | Metabolic weight loss of larvae when food is totally depleted | 0.016 |
| ***d_2_*** |  | 0.667 |

The original equations were calibrated at 26 ºC*.* The change in metabolic rate with temperature is described by the function *f*(*T_t_*), calculated as:

$f(T_{t})=f_{T}\frac{r(T_{t})-r(13.4 ^{^{\circ}}\text{C)}}{r(26 ^{^{\circ}}\text{C})-r(13.4 ^{^{\circ}}\text{C)}}$,

where *f_T_* is the value at 26 ºC (*f_T_* = 0.001). *r*(*T_t_*) is calculated according to an enzyme kinetics model , assuming that the rate of development is determined by a single rate-controlling enzyme which is reversibly denaturated at high and low temperatures. Skeeter Buster uses a simplified version of this model , which assumes inactivation only at high temperatures. 13.4 ºC is the lower developmental threshold at which *f*(*T_t_*) is set to zero.

*W*(*t*) and *F*(*t*) are calculated each day for each cohort in each container using Euler’s method with a resolution of 8 steps per day.

**References**

1. Magori K, Legros M, Puente ME, Focks DA, Scott TW, et al. (2009) Skeeter Buster: a stochastic, spatially-explicit modeling tool for studying *Aedes aegypti* population replacement and population suppression strategies. PLoS Negl Trop Dis 3: e508.

2. Gilpin ME, McClelland GAH (1979) Systems-analysis of the yellow fever mosquito *Aedes aegypti*. Forts Zool 25: 355-388.

3. Sharpe PJH, DeMichele DW (1977) Reaction kinetics of poikilotherm development. J Theor Biol 64: 649-670.

4. Schoolfield RM, Sharpe PJH, Magnuson CE (1981) Non-linear regression of biological temperature-dependent rate models based on absolute reaction-rate theory. J Theor Biol 88: 719-731.

5. Bar-Zeev M (1958) The effect of temperature on the growth rate and survival of the immature stages of *Aedes aegypti*. Bull Entomol Res 49: 157-163.
